# Chemoenzymatic Production and Engineering of Chitooligosaccharides and *N*-acetyl Glucosamine for Refining Biological Activities

**DOI:** 10.3389/fchem.2020.00469

**Published:** 2020-06-24

**Authors:** Manish Kumar, Meenakshi Rajput, Twinkle Soni, Vivekanand Vivekanand, Nidhi Pareek

**Affiliations:** ^1^Microbial Catalysis and Process Engineering Laboratory, Department of Microbiology, School of Life Sciences, Central University of Rajasthan, Ajmer, India; ^2^Centre for Energy and Environment, Malaviya National Institute of Technology, Jaipur, India

**Keywords:** chemoenzymatic, chitinase, ionic liquids, chitooligosaccharides, *N*-acetyl glucosamine, biological activities

## Abstract

Chitooligosaccharides (COS) and *N*-acetyl glucosamine (GlcNAc) are currently of enormous relevance to pharmaceutical, nutraceutical, cosmetics, food, and agriculture industries due to their wide range of biological activities, which include antimicrobial, antitumor, antioxidant, anticoagulant, wound healing, immunoregulatory, and hypocholesterolemic effects. A range of methods have been developed for the synthesis of COS with a specific degree of polymerization along with high production titres. In this respect, chemical, enzymatic, and microbial means, along with modern genetic manipulation techniques, have been extensively explored; however no method has been able to competently produce defined COS and GlcNAc in a mono-system approach. Henceforth, the chitin research has turned toward increased exploration of chemoenzymatic processes for COS and GlcNAc generation. Recent developments in the area of green chemicals, mainly ionic liquids, proved vital for the specified COS and GlcNAc synthesis with better yield and purity. Moreover, engineering of COS and GlcNAc to generate novel derivatives viz. carboxylated, sulfated, phenolic acid conjugated, amino derived COS, etc., further improved their biological activities. Consequently, chemoenzymatic synthesis and engineering of COS and GlcNAc emerged as a useful approach to lead the biologically-active compound-based biomedical research to an advanced prospect in the forthcoming era.

## Introduction

Modern biomedical research largely focuses on the biomaterials obtained from natural polymers (Raghavendra et al., 2015). Biopolymer-derived biomaterials indicate the tremendous possibility of utilization in the biomedical sector owing to their biocompatible and biodegradable nature. Among the biopolymers, chitin, and chitosan (CHS) have been extensively researched for their biological activities (Kumar, 2000). Chitin is a linear polysaccharide of β(1 → 4) linked *N*-acetyl-D-glucosamine (GlcNAc) monomers whereas CHS is a homo- or hetero-polymer of GlcNAc and D-glucosamine (GlcN) residues (Islam et al., 2017). The high MW and degree of polymerization (DP) limit the water solubility of these polymers. Water insolubility and high crystallinity limit industrial applicability of chitin and CHS, however, their transformation into soluble and more biologically active forms, i.e., chitooligosaccharides (COS) and GlcNAc, provides a way to overcome it (Qin and Zhao, 2019).

COS are the degraded products of chitin or CHS with DP ranging from 2 to 20. A wide range of biological activities, such as antimicrobial, anti-tumor, anti-oxidative, immunostimulatory, and anti-inflammatory activities, makes COS and GlcNAc molecules of interest for diverse industrial applications (Liaqat and Eltem, 2018). Despite having numerous significant applications, the production routes of COS and GlcNAc are still in their infancy in terms of bio-sustainability. Currently, chemical processes are predominantly used for the production of COS and GlcNAc; however, degradation of product quality, generation of undesirable byproducts, and environmental concerns limits their application (Varun et al., 2017). Moreover, interest has been raised over enzymatic conversion (employing chitinase and/or N-acetyl glucosaminidase) of chitin and CHS to COS and GlcNAc that has the advantage of producing defined products with high specificity (Kumar et al., 2018a). However, the limited availability of high enzyme yielding microbial resources, along with the high cost of enzyme/product extraction and purification bioprocesses and low yields, restricted the development of chitin bioconversion processes at a commercial scale. Therefore, the scientific community is currently focusing on the development of highly effective yet environmentally-friendly methods. In this regard, the synergistic approach of employing both chemicals and biocatalysts, i.e., the chemoenzymatic approach, seems to be more promising than other methods. The substrate pretreatment using eco-friendly chemicals, viz. ionic liquids, have shown remarkable possibilities to replace the traditionally-used harsh and toxic substances. Ionic liquids are being more frequently used in chitin research because of their high dissolving capacity, ease of handling, reusability, and lower toxicity (Li et al., 2019). The application of a mild chemical treatment contributes to increased vulnerability of chitin and CHS for further enzymatic action and thus is beneficial in enhancing the quality and yield of COS and GlcNAc. Additionally, transglycosylation methods are also in use for the synthesis of COS. The enzymes and chemicals with the transglycosylation ability can synthesize COS with a high DP, more purity, and more affinity (Alsina et al., 2019; Bhuvanachandra and Podile, 2020). The biological activities of COS depend upon DP, MW, and the pattern of acetylation (PA)/deacetylation (PD). The engineering of COS and GlcNAc to enhance their biological activities is possible due to the employment of developments made so far in the field of biotechnology and chemistry. Moreover, the medicinal applications of COS and GlcNAc can be improved by altering the chemical structure, which in turn increases their biological motion (Ngo et al., 2019). Considering the recent advancements in the biomedical applications of COS, GlcNAc, and their derivatives, this review mainly focuses on the bioconversion production approaches to enhance cost-effective production in a green manner. Moreover, the review also enlightens the developments made in the engineering and derivatization of COS and GlcNAc to expand their biological functions.

## COS and GlcNAc

The commercial application of chitin can be augmented by overcoming its high crystallinity and insolubility. It can be achieved through the interconversion of chitin into COS and GlcNAc. COS are the homo- or heteropolymers of GlcNAc and D-glucosamine (GlcN) units in varying proportions, and are generated via chitin degradation (Aam et al., 2010). The average MW of COS is <39 kDa with <20 DP (Mourya et al., 2011). GlcNAc is an essential factor in hyaluronic acid and keratin sulfate present on the cell surface (Chen et al., 2010). GlcNAc is also explored as a renewable resource for ethanol production. COS and GlcNAc can be synthesized by employing either physical, chemical, or biological methods alone or in a synergistic manner (Kumar et al., 2018a). The demand for COS and GlcNAc has tremendously increased due to their vast potential applications in biomedical, food, cosmetic, and agricultural sectors. The biological activities (antimicrobial, anticancer, antioxidant, immune-stimulating activity, etc.) of COS and GlcNAc largely depends on the DP and sequence of acetylated and deacetylated units (Halder and Mondal, 2018) along with the solubility that increases with DP in an inversely proportional manner (Liaqat and Eltem, 2018). Water solubility and lower viscosity of COS are associated with their chain lengths and free amino groups in GlcN units. COS are usually insoluble in acetone, butanol, ethanol, ethyl acetate, propanol, and pyridine, whereas they are soluble in water and partially soluble in methanol and dimethyl sulfoxide (Mourya et al., 2011).

## Biological Activities of COS and GlcNAc

**Figure 1:**
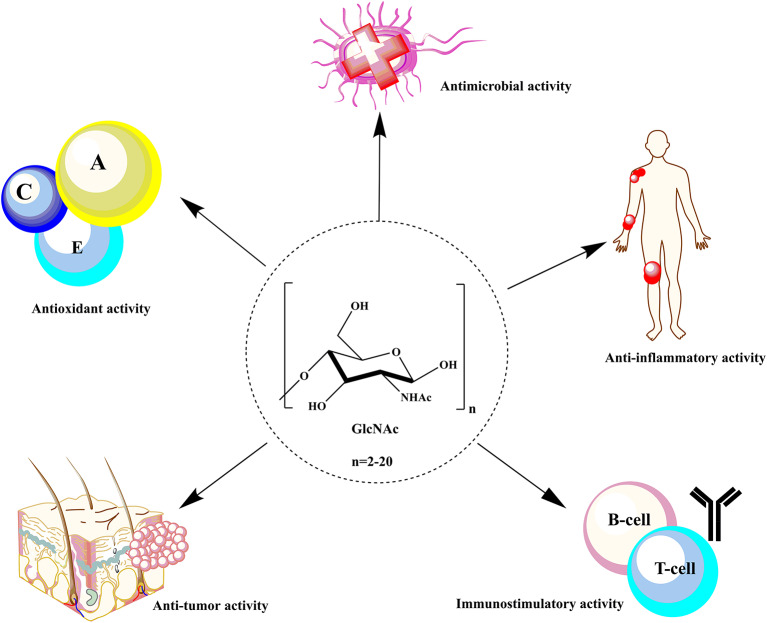
Biological activities of COS and GlcNAc in the biomedical sector.

The water solubility and low MW of COS and GlcNAc enhance their applicability in food and agricultural sectors. Still, the most significant and extraordinary applications of COS and GlcNAc are in human healthcare. Several biomedical applications of COS have been reported, such as forvectors in gene delivery, to prevent tumor growth, asthma treatment, improvement of bone strength, and fabrication of tissue scaffolds (Aam et al., 2010; Nagpure et al., 2014; Chen and Zhao, 2019) (Table 1). The wide range of applications of COS and GlcNAc are owed to their remarkable biological activities viz. antimicrobial, anti-tumor, antioxidant, immunoregulatory, blood pressure control, and hypocholesterolemic effects (Liaqat and Eltem, 2018) (Figure 1). The biological activities of COS largely depend upon DP and DA. The mechanism underlying the biological activities of COS is still not well-studied due to a lack of purity, quality, and proper characterization (Zou et al., 2016). The presence of primary amino groups have been reported to contribute in the antimicrobial activity of COS and GlcNAc. COS have been known to accomplish microbial cell death by modifying the permeability of the cell membrane. Generally, COS are positively charged, which enables them to bind and adsorb readily on the negative cell wall that leads to DNA rupture and blocking of RNA transcription (Mei et al., 2015).

**Table 1:** Biological activities of COS and GlcN.

**COS and GlcN**	**Biological activities**	**Experimental model**	**Techniques**	**Satisfactory outcomes**	**References**
COS mixture [(GlcNAc)_4−11_]	Antifungal	*Trichophyton rubrum* Hartley guinea pigs	Broth microdilution assay Agar diffusion assay	Minimal inhibitory concentration (MIC) of COS: 0.25–0.5% Inhibition rate at COS concentration 0.1% −1.0%: 13.04%−46.38%	Mei et al., 2015
COS micture [(GlcNAc)_2−6_]	Antioxidant	High fat diet mouse	Superoxide radical scavenging activity Hydroxyl radical-scavenging assay DPPH radical scavenging	Scavenging ability at COS concentration 5 mg/mL: Superoxide radical: 32.5% Hydroxyl radical: 41.9% DPPH radical: 89.1%	Qu and Han, 2016
Four COS group based on MW: I: 100 kDa II: 100–10 kDa III: 10–1 kDa IV: <1 kDa	Antioxidant Antiproliferative	Carcinoma cells (HEPG2, HCT, MCF7 cells)	DPPH method ABTS radical cation decolorization assay Ferric reducing antioxidant power (FRAP) method Sulforhodamine B assay	Free radical scavenging ability group I-IV (μg TE/mg COS): DPPH: 1.84–86.97 ABTS: 1.99–93.56 Reducing power FRAP: 1.44–91.07 IC_50_ values of group II (μg/mL): HEPG2-1.56; HCT- 1.84; MCF7- 2.20	El-Sayed et al., 2017b
Chitosan (300 kDa) Four COS group based on MW: I: 100 kDa II: 100–10 kDa III: 10–1 kDa IV: <1 kDa	Antimicrobial	*Bacillus substilis* *Staphylococcus aureus* *Pseudomonas aeruginosa* *Candida albicans* *Saccharomyces chevalieri*	Filter paper disc method	Chitosan -inhibition zone diameter (mm): *B. substilis*-12 *S. chevalieri*-20 Group IV COS inhibition zone diameter (mm): *C. albicans-*15	El-Sayed et al., 2017a
(GlcNAc)_5−12_	Antiproliferative	Human cervical cancer cell lines Human endometrial cancer cell lines Human breast cancer cell lines	MTT assay	IC_50_ of Human cervical cancer cell line (C33A) (mg/ml) at: 24 h-5.54 48 h-3.44 72 h-2.43	Zhao et al., 2019

Similarly, the amino group also plays a key role in the antioxidant activity of COS. The amino group stabilizes unstable free radicals that results in an interrupted radical chain reaction (Zhao et al., 2013). In a recent study, Bai et al. (2020) developed selenium nanoparticles loaded CHS/COS microparticles and investigated their ability to alleviate the oxidative stress induced by alcohol in mice. During oxidative stress, the concentration of thiobarbituric acid reactive substances (TBARS) and total carbonyl compounds (TCC) increases with the decrease in superoxide dismutase (SOD), catalase (CAT), glutathione (GSH), and glutathione peroxidase (GSHpx) levels in the cell. The study conducted an acute lethal test and 50% ethanol challenge to observe the efficiency of the developed microparticles as a proficient antioxidative agent, and they observed a rise in SOD, CAT, GSH, and GSHpx with a reduction in TBARS and TCC levels that proved the antioxidant potential of COS. In the present decade, the anti-tumor activity of COS and GlcNAc has been under extensive exploration by several researchers (Wang et al., 2019; Wu et al., 2019). Yu et al. (2016) conjugated a short synthetic peptide ES2 (Endostatin2) with soluble O-(2-hydroxy) propyl-3-trimethylammonium COS chloride (HTCOSC) through covalent binding and reported the considerable anti-angiogenic potential of the conjugate. Wang et al. (2019) further examined the role of this conjugate in inhibiting tumor growth in tumor-bearing C57BL/6 female mice by affecting vascular endothelial growth factor (VEGF), microvessel density (MVD), and caspase-3 through immunohistochemistry techniques. The results of the study revealed that the conjugate had successfully blocked cell proliferation in endothelial cells by arresting the cell cycle at the G0/G1phase. Furthermore, the study also suggested that the expression of caspase-3 was upregulated with a decrease in the number of microvessels in tumors. COS can also reduce the azomethane and dextran sulfate sodium induced by colorectal cancer in mice by restoring the composition of the gastrointestinal fungal community by increasing the healthy microbes (prebiotics) and reducing pathobionts, as alterations in the gastric and intestinal microbiota were observed during colorectal cancer (Wu et al., 2019). Thus, recent studies suggest that COS can serve as a compelling contender for the treatment of cancer. However, the mechanism behind the anti-tumor activity of COS is largely unknown. Additionally, selective electrostatic interactions with the tumor cell, MW, DP, immunostimulating, and antiangiogenic effects are also considered to be responsible for the anti-tumor activities of COS and GlcNAc (Liaqat and Eltem, 2018).

The anti-inflammatory activities of COS have been shown to mainly depend upon their physicochemical properties. COS with lower DP shows better antioxidant as well as anti-inflammatory activities (Santos-Moriano et al., 2019). The study conducted by Santos-Moriano et al. (2019) analyzed the anti-inflammatory activity of a mixture of COS produced by various combinations of enzymes and substrates on murine macrophages (RAW 264.7) by monitoring the concentration of TNF-α (tumor necrosis factor-α) through a TNF-α ELISA kit. They observed that after stimulation by lipopolysaccharides, TNF-α concentration was decreased and the highest TNF-α concentration of 1,575 pg ml^−1^ was achieved after post-stimulation of 6 h with the application of a 10 ng mixture of lipopolysaccharides. The reported remarkable biological activities of COS and GlcNAc intensified the demand for their commercial production. Therefore, the present era of chitin research is mainly focused on the development of an efficient process for the production of high COS and GlcNAc titres with improved biological activities. The following section presents an insight into the current developments being made and the diverse strategies employed for COS and GlcNAc production.

## Strategies for COS and GlcNAc Production

**Figure 2:**
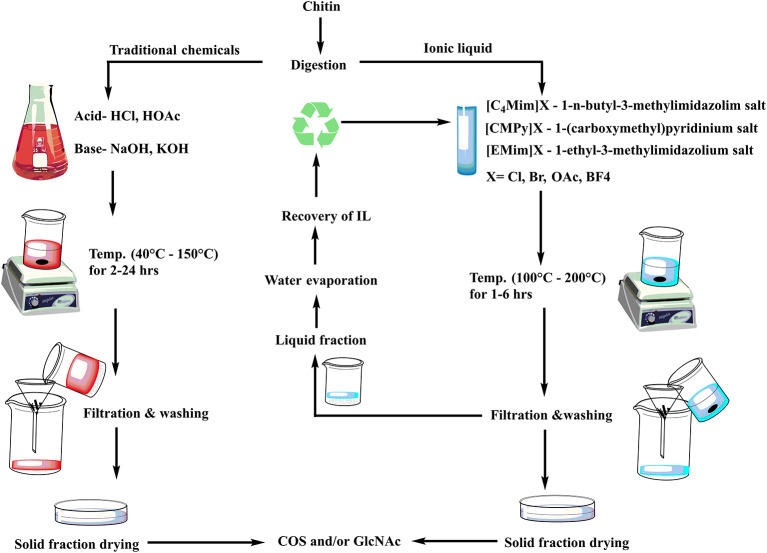
Different approaches for COS and GlcNAc production.

Over the last few decades, the main issue in chitin research has been to develop an efficient and sustainable process for COS and GlcNAc production. Several approaches envisaging chemical and biological means are in practice for COS and GlcNAc production (Figure 2). Several chemical-based methods have been used for COS generation, and among them, acid hydrolysis of chitin is the most common (Novikov, 2004; Jung and Park, 2014). However, the acid-based production of COS and GlcNAc has several disadvantages, such as the harsh reaction conditions, formation of unstable products, and environmental concerns (Jung and Park, 2014). These issues can be addressed by the involvement of enzymatic conversion methods that allow for better reaction control with improved biological activities (Liang et al., 2018). However, commercial employment of enzymatic conversion has been restricted due to relatively low production levels and a high extraction cost. Therefore, researchers are focusing on the development of synergistic chemical treatments and enzymatic hydrolysis processes for chitin degradation with special consideration on the utilization of non-toxic or less toxic chemicals (Nu et al., 2017). The chemoenzymatic approach showed high potentiality of COS and GlcNAc production from chitin. Moreover, the transglycosylation approach for the synthesis of long-chain COS has also illustrated noteworthy prospects for industrial applications (Sinha et al., 2016). The various production methods for COS and GlcNAc have been summarized in Table 2 with their respective advantages/disadvantages and possible industrial implementations. The forthcoming section exemplifies the recent developments in enzymatic, chemical, and chemoenzymatic production strategies for COS and GlcNAc generation.

**Table 2:** Summary of various production methods of COS and GLcNAc.

	**Enzymatic**	**Chemical**	**Microbial engineering**	**Transglycosylation**	**Chemoenzymatic**
Reaction conditions	Gentle (Temp.: 37–60°C pH: 3–12)	Harsh (Conc. acid Temp.:40–200°C for 1–24 h)	Gentle (Temp: 40–60°C pH:5.5–12)	Gentle (Temp.: 40- 60°C pH:5–7)	Mild (Temp.: 100–200°C for 1–6 h)
Product yield	Low	High	High	Low	High
Specificity	High	Low	High	High	High
Toxicity	Non-toxic	High	Non-toxic	Non-toxic	Low to moderately toxic
Possibility of industrial implementation	Limited	High but separation of product is costly	High	Limited	High

### Enzymatic Approach

Over the last few decades, significant advancements have been made in the enzymatic methods of chitin biotransformation into COS and GlcNAc and these have emerged as a promising substitute to chemical methods. Chemical conversion is associated with various disadvantages, i.e., generation of toxic compounds, alteration in original chemical structure, environmental pollution, variable product quality, and low product yields. Whereas, the enzymatic mean of conversion has an edge due to its eco-friendly approach with ease of control. Chitozymes have been considered as the major group of enzymes involved in chitin transformations. Among the group, chitinases (E.C 3.2.2.14) are the key biocatalysts explored for the enzymatic production of COS and GlcNAc (Table 3). These glycosyl hydrolases degrade chitin into low/high MW COS and GlcNAc. Chitinases research has gained attention after the discovery of their remarkable role as a biocontrol agent against fungal phytopathogens and harmful insects in plant defense systems. Based on the cleavage patterns, chitinases can be classified into three major groups: i.e., (a) exo-chitinases, (b) endo-chitinases, and (c) β-*N*-acetylglucosaminidase (Patil et al., 2000; Hamid et al., 2013). The enzymatic production of COS using chitinases needs high levels of endo-chitinases, whereas GlcNAc production requires exo-chitinases and β-N-acetylglucosaminidase in a high quantity (Lombard et al., 2014). Depending on the amino acid sequence similarity (www.cazy.org), chitinases are placed into glycosyl hydrolase (GH) families 18, 19, and 20. The ground of this categorization comprises of *N*-terminal sequence, enzyme localization, signal peptide, isoelectric pH, and inducers. Bacterial and fungal chitinases belong to GH family 18 whereas GH family 19 primarily encompasses plant chitinases (Kumar et al., 2018c). Depending on the degree of acetylation of chitosan, chitinases also possess the ability to degrade chitosan (Hartl et al., 2012). To date, various studies have been reported on COS and GlcNAc production utilizing chitinases. Zhang et al. (2018) developed a novel recombinant of chitinase gene CmChi1 derived from *Chitinolyticabacter meiyuanensis* SYBC-H1 by expressing it in *Escherichia coli* BL21 (DE-3) cells. The (GlcNAc)_2−6_ and colloidal chitin (CC) were utilized as substrates where the recombinant enzyme showed 15.3 U mg^−1^ activity with CC. The enzyme displaying the exo- cleavage pattern as (GlcNAc)_2_ was produced as the main product from both the substrates, whereas the considerable release of GlcNAc and (GlcNAc)_3−4_ in small amounts revealed the endo-pattern of cleavage. Moreover, weak β-*N*-acetylglucosaminidase activity was also observed, thus recombinant CmChi1 chitinase appeared as a possible candidate to achieve an 100% yield of GlcNAc from chitin. Similarly, a recombinant of the chit42 gene from *Trichoderma harzianum* was developed in the *Pichia pastoris* expression system (42 kDa, 150 mU) (Kidibule et al., 2018). The recombinant enzyme was then utilized for COS and GlcNAc production using CC and CHS as the substrates and observed to produce (GlcNAc)_1−2_ and GlcNAc as the major products (Kidibule et al., 2018).

**Table 3:** Enzymatic production and analysis techniques of COS and GlcNAc.

**Enzyme**	**Source**	**COS and GlcNAc**	**Analysis**	**References**
Chitinase (20–120 kDa)	*Chitinibacter* sp. GC72	GlcNAc	TLC	Gao et al., 2015
Chitinase (35 and 50 kDa)	*Lecanicillium lecanii*	(GlcNAc)_2−5_	MALDI-ToF	Villa-Lerma et al., 2016
Chitinase	*Chitinolyticbacter meiyuanensis SYBC-H1*	GlcNAc	HPLC ESI-MS	Zhang et al., 2016
Chitinase (67 kDa)	*Paenicibacillus barengoltzii CAU904*	(GlcNAc)_2−4_ GlcNAc	TLC HPLC	Fu et al., 2016
Chitinase (74 kDa)	*Bacillus thuringiensis*	(GlcNAc)_2_ GlcNAc	HPLC	Honda et al., 2017
Chitinase (69 kDa)	*Paenibacillus pasadenensis CS0611*	(GlcNAc)_2_	TLC HPLC	Guo et al., 2017
Chitinase (94.2 kDa)	*Salinivibrio* sp. *BAO-1081*	(GlcNAc)_2_	TLC HPLC	Le and Yang, 2018
Chitinase	*Esherichia fergusonii*	GlcNAc	TLC HPLC	Kim et al., 2018
Chitinase (12.8 kDa)	*Streptomyces thermocarboxydus TKU045*	(GlcNAc)_1−6_	MALDI-ToF HPLC	Tran et al., 2019

Thermo-alkali stable, extracellular chitinase (10.5 kDa) was purified from *Streptomyces chilikensis* RC1830 isolated from brackish lake water sediment (Ray et al., 2019). The study suggested that purified enzymes possess a high binding affinity with CC as compared to starch, xylan, and carboxymethyl cellulose and have a significant conversion efficiency at 60°C (pH 11). High acid tolerance and thermal stability are considered as the desired characteristics of the enzymes to be utilized for COS and GlcNAc production at an industrial level. A 46kDa Chi1 was purified from *Streptomyces thermodiastaticus* HF 3-3 with specific activity of 2.4 U mg^−1^. The enzyme was found to be remarkably stable over a wide range of pH, temperatures, and chemical exposures (Take et al., 2018). Moreover, thin-layer chromatography (TLC) analysis of CC cleavage products revealed that Chi1 exhibited endo- pattern of cleavage with the generation of (GlcNAc)_2_ as the major product and minimal amounts of (GlcNAc)_3_ and GlcNAc (Take et al., 2018). Extracellular chitinases production and its utilization for COS and GlcNAc conversion has also been extensively studied, and some findings have shown high potentiality in terms of catalytic efficiency and/or thermal and pH stability (Halder et al., 2019). A 50 kDa chitinase from thermophilic *Humicola grisea* displayed significant catalytic efficiency toward CC with the generation of GlcNAc and COS with DP of 2 and 3 (Kumar et al., 2018b). The enzyme showed optimum enzyme activity at a pH of 3.0 and a temperature of 70°C. Another study reported a thermostable chitinase Chi1 homologously produced from *Myceliophthora thermophila* C1 with high thermostability and activity toward chitin, CHS, modified CHS, and chitin oligosaccharides (Krolicka et al., 2018). The study reported that Chi1 has notable stability at 40°C (90%, 140 h) and 50°C (90%, 168 h).

### Chemical Approach

**Figure 3:**
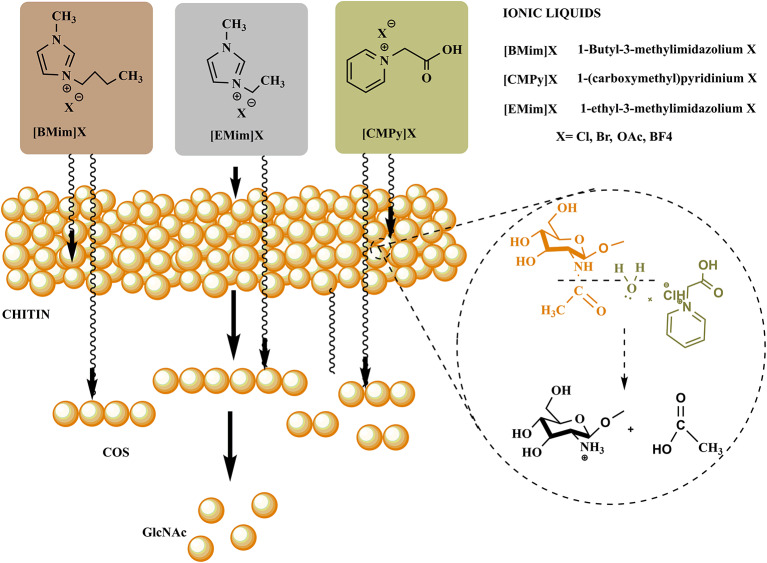
Conventional and emerging green chemicals based production steps of COS and GlcNAc.

The chemical methods for COS and GlcNAc production are most frequently applied for commercial-scale production through shrimp and crab shells. The extraction is carried out by acid hydrolysis and oxidative degradation methods. The chemical conversion of chitin usually employs hydrochloric acid, sulphuric acid, acetic acid, lactic acid, trichloroacetic acid, and formic acid (Hamed et al., 2016). Processing temperature, time, and concentration of acid are vital factors that affect the rate of conversion. The polymer conversion research is presently aimed at utilizing relatively less toxic chemicals viz. ionic liquids to compete with the production levels as well as to overcome environmental safety issues (Figure 3).

#### Conventional Chemicals

The practice of utilizing harsh chemicals for the degradation of chitin has been longstanding. A three-step method for acid hydrolysis of chitin was reported by Falk et al. (1966). The study showed chitin hydrolysis using concentrated hydrochloric acid in the stages including: (a) production of smaller polymeric units or oligomers, (b) output of GlcNAc from the latter, and (c) GlcNAc conversion into glucosamine and acetic acid. GlcNAc was usually obtained by acid hydrolysis of chitin at lower temperatures or by acetylation reaction of GlcN with acetic anhydride, whereas GlcN is the product of chitin acid hydrolysis at high temperatures (Mojarrad et al., 2007). Although production of GlcNAc through chemical hydrolysis appeared economically reasonable, limited specificity of chemical catalysts, low product yield, high acidic waste generation, and operational costs demotes the application of chemical methods. Mechanocatalytic depolymerization of chitin was reported, in which soluble chitin oligomers were produced through the grounding of chitin with sulfuric acid in a ball mill and further hydrolysis at high temperature with additional acid resulted in the GlcNAc production (Yabushita et al., 2015). Similarly, an advanced approach for hydrolysis of chitin into GlcNAc and GlcN with significantly reduced amounts of acid as catalysts was demonstrated by Zhang and Yan (2017). The researchers employed co-solvents, including several aprotic solvents and etheric solvents, resulting in an 80% GlcN yield in the presence of 100 mM sulfuric acid (175°C, 60 mins). Recently, sulphuric acid hydrolysis of straw mushroom was conducted and a yield of 56.8132 mg g^−1^ glucosamine was achieved (Zhang and Sutheerawattananonda, 2020). Although the traditional chemicals are quite useful in terms of production levels, in view of product quality and environmental safety, it has become imperative to develop an alternative approach.

#### Ionic Liquids

Solvents composed of volatile organic compounds (VOC) tops the list of dangerous chemicals. Volatile organic solvents act as primary reaction media for the production of various chemicals at an industrial scale and proved to be harmful to both the environment and to worker's health. Moreover, the extra cost required for the disposal of these VOCs, recyclability issues, and their separation from the desired reaction product make them highly undesirable (Mallakpour and Dinari, 2012). Therefore, a viable and environmentally-friendly alternative for volatile organic solvents is required. Ionic liquids (ILs) serve as an attractive alternate capable of overcoming all the limitations of traditional volatile organic solvents. ILs can be defined as salts with a melting point less than the boiling point of water (Wilkes, 2004). ILs are mainly composed of ions, i.e., organic cations and inorganic/organic anions (Vekariya, 2017). The highly promising properties of ILs include thermal stability, non-flammability, secure containment, and easy recycling, and have attracted the attention of many researchers in the last two decades (Marcus, 2016). The electrostatic forces lying inbetween the ions are responsible for the stability, non-volatility, and non-flammability of ILs. The ILs are renowned as “green solvents” because of their non-volatile nature and low vapor pressure (MacFarlane et al., 2014). Moreover, they are also designated with several other names, viz. designer solvents, molten salts, neoteric solvents, and ionic fluids. ILs are regarded as “designer solvents” because the polarity and hydrophilicity/hydrophobicity can be designed by various suitable combinations of cation and anions (Passos et al., 2014). Another advantage of ILs is that, during the reaction, they do not require harsh conditions and respond within a relatively short period (Maier et al., 2017).

**Figure 4:**
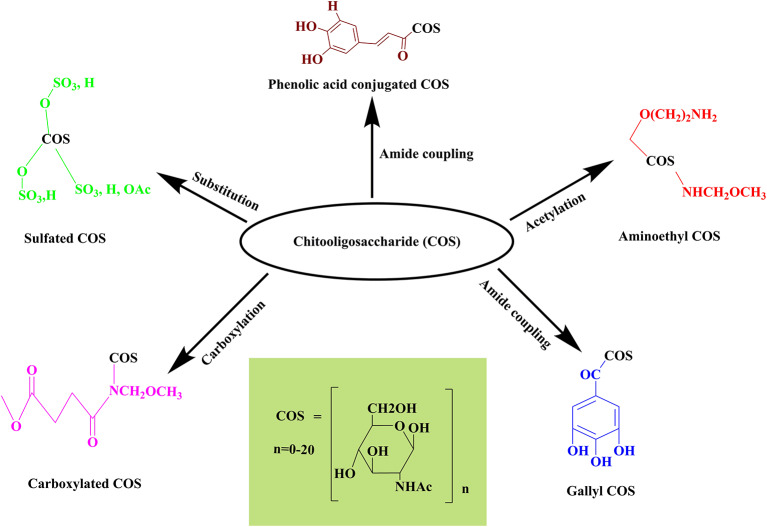
Representation of the mechanism involved in COS and GlcNAc generation through ionic liquid.

ILs have been explored widely for the production of various valuable materials and chemicals by utilizing lignocellulosic biomass (Yoo et al., 2017). However, the utilization of ILs for the processing of chitin to yield valuable products (GlcNAc, COS, CHS, and other chemicals) is still in its infancy. The utilization of ionic liquids provided a sustainable way to process chitin in the green chemistry realm for the generation of defined products with novel biological properties. ILs firstly disrupt the intra- and intermolecular hydrogen bonding in the polysaccharides (chitin, chitosan, and COS) followed by the formation of new hydrogen bonds between the anions of ionic liquids and hydroxyl groups of polysaccharides. It leads to the formation of amorphous chitin having a hydrated gel-like structure with relatively lower crystallinity. The polymeric chain of chitin gets decrystallized due to the disruption of hydrogen bonds with increased accessibility to reactants (Figure 4). Thus, for chitin processing, ILs can either be employed during pretreatment or as a co-solvent/solvent in simultaneous treatment (Shamshina and Berton, 2020).

Several studies have been reported on the employment of ionic liquids ([C_2_mim][OAc] and [C_2_mim][MeO(H)PO_2_]) for the pretreatment of chitin prior to the enzymatic hydrolysis for the efficient production of COS and GlcNAc (Table 4). Yu et al. (2015) compared the degradative effects of modified cellulase (Cell-ALD 10k) on chitosan in IL [Gly]BF4 and acetic acid HAc systems. The FTIR spectra revealed that [Gly]BF4 has successfully disrupted the inter- and intramolecular hydrogen bonding in chitosan more effectively than HAc. They evidenced 76.36% yields of COS at optimal reaction conditions (pH 5, 50°C). Furthermore, the researchers also reported a significant decrease in the crystallinity of chitosan with the increase in free amino groups by employing [Gly]BF4. Gillard et al. (2016) made an attempt for the generation of glucosamine containing oligomers by glycosylating ionic linker tag (ITag) grafted acceptors with thioglucosamine donors by the β-(1 → 4) linkage. For this, they opted for the ionic catch-and-release oligosaccharide synthesis (ICROS) methodology. The abovementioned studies reflected the vast potentiality of ILs in the production of COS and GlcNAc. However, further research is required to enhance the recyclability and reusability of ILs along with the synergistic action with enzymatic methods.

**Table 4:** Ionic liquids employed for COS and GlcNAc production.

**Ionic liquids**	**Ionic liquid function**	**COS and GlcNAc production process**	**Products obtained**	**References**
[HOSO_2_C_3_mim][CF_3_SO_3_] [HOSO_2_C_8_mim][NTf_2_]	Catalyst	Acid hydrolysis	GlcNAc	Pischek et al., 2014
[C_2_mim][OAc] [C_2_mim][MeO(H)PO_2_]	Pretreatment Co-solvent	Enzymatic hydrolysis	GlcNAc (GlcNAc)_2_	Husson et al., 2017
[C_2_mim][OAc]	Chitin extraction	Enzymatic hydrolysis	GlcNAc (GlcNAc)_2_ (GlcNAc)_3_	Berton et al., 2018
[BMIm]Ac	Pretreatment	Enzymatic hydrolysis	GlcNAc (GlcNAc)_2_	Xu et al., 2018
[C_2_mim][OAc]	Pretreatment	Enzymatic hydrolysis	GlcNAc (GlcNAc)_2_	Li et al., 2019

### Microbial Engineering Approach

The enzymatic route of COS and GlcNAc production mainly exploits chitinolytic enzymes, that are obtained from a range of bacteria, fungi, and plants i.e., *Serratia marcescens, Bacillus circulans, Streptomyces griseus, Saccharomyces cerevisiae, Candida albicans, Neurospora crassa, and Trichoderma harzianum* (Hamid et al., 2013). However, the production of enzymes with low activity, yield, and specificity encumbered the employment of these biocatalysts for large-scale production of COS and GlcNAc. Thus, to increase the expression level of chitinolytic enzymes from microbial sources, researchers are focussing on the engineering of microbial cells by using different heterologous expression systems (Pan et al., 2019). To date, *Escherichia coli* (Yang et al., 2016; Abdel-Salam et al., 2018) and *Pichia pastoris* (Ueda et al., 2017; Menghiu et al., 2019) have been explored widely for the expression of chitinases from distinct sources. However, the heterogeneous expression of enzymes in these systems holds many challenges, including the formation of inclusion bodies. The eukaryotic *Pichia pastoris* expression system also possesses several limitations, including its complex nature, low expression levels, and extended incubation periods. Furthermore, the expression of intracellular enzymes faces more hurdles as they need the physical destruction of cells to secrete out, which may led to an increased cost of enzyme purification, low activities, and enzyme inactivation. Thus, it is undeniably necessary to select an appropriate expression host system along with genetic engineering for the development of potent strains capable of producing high chitinase titres with notable catalytic efficiency to convert chitin into COS and GlcNAc. Moreover, the genomic engineering of microbial cells has also proven to be a favorable approach for obtaining COS with better yields to support research and applications in the clinical and food industry (Ruffing et al., 2006).

Penta-*N*-acetyl-chitopentaose mixture has been produced by cultivating recombinant *E. coli* cells expressing nodC gene from *Mesorhizobium loti* (Zhang et al., 2007). The study developed a two-step fermentation process to improve the COS productivity in recombinant *E. coli* cells and observed significant enhancement in COS yield and production up to 930 mg l^−1^ and 37 mg l^−1^h^−1^, respectively, in a 10-L bioreactor. In *Bacillus subtilis WB600*, the addition of signal peptide NprB has significantly increased the extracellular expression of chitinase (Chisb) from 2.28 to 35.54 U mL^−1^ (Pan et al., 2019). Chisb was purified with HisTrapHP and showed a 3.06-fold purification with 141.43 U mg^−1^ specific activity when colloidal chitin was used as a substrate. Furthermore, the study reported that the ribosome binding sites (RBS) optimized with spacer sequences and molecular docking technology in combination with site-directed mutagenesis increased the expression level and the specific activity of Chisb. Chitinase activity with 20 RBS sites (R1-R20) was analyzed, and R13, R19, and R20 have reported to considerably augment the enzyme activity by 45.39, 36.83, and 14.77%, respectively. The substrate specificity of Chisb was found to be highest with (GlcNAc)_5_ as 340.76 U mg^−1^ among colloidal chitin, powdery chitin, chitosan, pNP-GlcNAc, and (GlcNAc)_2−4_. Deng et al. (2019) performed whole-genome sequencing of *Corynebacterium glutamicum S9114* to evaluate the function of different genes in glutamate and GlcNAc synthesis pathways. They observed that blocking the expression of nagA (GlcNAc-6-phosphate deacetylase) and gamA (GlcN-6-phosphate deaminase) in *C. glutamicum* S9114 increased the production of GlcNAc by 54.8% from 3.1 to 4.8 gL^−1^. Further, the silencing of ldh (lactate dehydrogenase) gene led to the inhibition of lactate synthesis, which resulted in increased GlcNAc titer value, i.e., 5.4 gL^−1^. Moreover, a recombinant CGGN-*GNA1- CgglmS* has been developed by expressing a vital gene for GlcN synthesis i.e., glmS from various sources in vector pJYW-4-ceN to rise the GlcNAc titer value (6.9 gL^−1^). Recent advancements in microbial engineering have shown a high proficiency for enhanced production of chitinolytic enzymes that can further be effectively explored for COS and GlcNAc production. However, the commercialization of engineered strains needs a lot more research.

### Transglycosylation Approach

Recently, the chitin and CHS modifying enzymes have gained considerable attention as efficient tools for the production of well-defined COS and their derivatives. For this, genetically engineered chitinolytic enzymes possessing transglycosylation (TG) activity have emerged as exceptional tools. Transglycosylation and hydrolysis are two diverse and essential routes that are accomplished by chitinolytic enzymes. Transglycosylation activity involved the formation of a glycosidic bond amidst two saccharides by the relocation of sugar moiety from a suitable donor to an acceptor (Ling et al., 2018). Several chitinolytic enzymes exhibiting hydrolytic activity also possess transglycosylation ability to some extent. These abilities of chitinolytic enzymes help to determine their aptness in performing specific functions and applications. Hydrolysis generally led to the synthesis of monomeric or dimeric glucosamine units with low DP. In contrast, TG is a kinetically controlled process that results in the synthesis of size- and stereo-specific oligomers of chitin/CHS and their derivatives (Purushotham and Podile, 2012). The occurrence of TG activity during the hydrolytic process may results in the production of astonishing enzymatic products as it interferes with the hydrolytic activity of chitinases. Therefore, chitinases must be engineered in favor of hydrolysis or TG to get the desired outcome that meets the specific applications. Also, the genetically engineered chitinases with high TG activity and relatively reduced hydrolytic activity produce COS with high DP that can be utilized in the food and pharmaceutical industries.

COS with higher DP can be developed from COS with lower DP by using chitinolytic enzymes viz. chitinase, chitosanase, and other glycosidases exhibiting TG activity. For instance, Hattori et al. (2012), reported lysozyme-mediated TG for the production of COS with DP 6 to 15 by using β-1,4-(GlcNAc)_3_ as substrates. A hyper transglycosylating chitinase (*Ec*Chi1) with an endo-acting cleavage pattern was purified from *Enterobacter cloacae* subsp. *cloacae* 13047 (Mallakuntla et al., 2017). The (GlcNAc)_3−6_ were utilized as substrates for the production of COS via TG and resulted in the formation of products with DP ranging from 4 to 9. The study further concluded that the length of the COS substrate and the concentration of the enzyme significantly affects the TG activity of *Ec*Chi1. Similarly, a salt-tolerant chitinase B (FjChiB) was isolated from *Flavobacterium johnsoniae* UW101; it was also found to be exhibiting TG activity as it was utilizing (GlcNAc)_5_ and (GlcNAc)_6_ as the substrates for COS synthesis (Vaikuntapu et al., 2018). An N-acetylglucosaminidase derived from *Lecanicillium lecanii* on a submerged culture showed up both hydrolytic and TG activities. Findings signified that the enzyme was able to produce COS with DP from 2 to 6 units (Rojas-Osnaya et al., 2019). Recently, Bhuvanachandra and Podile (2020) expressed a *Cs*Chil gene from *Chitiniphilus shinanonensis* in *E. coli* and assessed its hydrolytic and TG ability. They found that production of (GlcNAc)_4_ was most effective when using (GlcNAc)_2_ as substrate. (GlcNAc)_4−6_ were also used as substrate but no higher chain production was observed. TG of (GlcNAc)_4_ resulted in the production of products with DP 2 to 6 where COS with DP 5 and 6 were less in fraction, as after 30 min of reaction the products were further hydrolyzed into shorter COS. Sirimontree et al. (2014) developed several mutants of chitinase A derived from *Vibrio harveyi* (*Vh*ChiA) for the production of longer COS through TG. However, the product formed by enhancing the TG activity is not always a longer chain COS; occasionally, disintegration could result in the generation of COS with less DP. HPLC analysis of the TG products formed by mutants W570G and D392N using GlcNAc as substrate showed improved TG efficiency but products immediately hydrolyzed into shorter COS. Contrarily, the products formed by mutants D313A and D313N were not hydrolyzed further and resulted in the accumulation of longer chain COS. This indicated that some mutational strategies should be adopted along with the enhancement of TG activity to prevent the decomposition of the product formed.

### Chemoenzymatic Approach

The production of COS and GlcNAc has been extensively explored using chemical and enzymatic processes, but both are associated with certain limitations. In the case of synthetic production, product quality and the environment is compromised, whereas enzymatic methods result in a low production yield. So, to fulfill the demand for commercial production, interest has been raised to develop synergy between different processes. In this context, the chemical pretreatment followed by enzymatic hydrolysis has illustrated significant enhancement in COS and GlcNAc production (Trung et al., 2020). Pretreatment of chitin before enzymatic hydrolysis not only decreases the crystallinity of the polymer but also makes the substrate readily accessible to the enzyme. In recent years, the solution of alkali (LiOH/NaOH/KOH)/urea as a solvent has also emerged as a highly efficient and comparatively eco-friendly solvent for the dissolution of chitin and cellulose by freeze-thaw methods (Xiong et al., 2013). However, Gong et al. (2016) conducted a study on the dissolution of chitin using a solvent comprised of aqueous solutions of KOH (8.4–25 wt%) /urea and through NMR analysis observed that chitin displayed appreciably good solubility (80%) through the freeze-thawing process. The study supported the dissolution power of alkali-based solvent systems in the order KOH > NaOH > LiOH. Recently, Sivaramakrishna et al. (2020) used KOH and solvent of KOH-urea aqueous solutions for the pretreatment of α-chitin before enzymatic hydrolysis with *Enterobacter cloacae* subsp. *cloacae* derived chitinase (*Ec*Chi1). The hydrolytic activity of KOH and KOH-urea pretreated α-chitin was increased significantly (91%) when compared to colloidal chitin and untreated α-chitin. Furthermore, the HPLC analysis of hydrolytic products obtained with treated α-chitin showed the generation of COS with DP3 along with DP1 and DP2, whereas with the colloidal chitin and untreated α-chitin only DP1and DP2 COS were produced. In another study, the chemo-thermal pretreatment of chitin by using H_2_SO_4_ (2% w/v) at 21°C and 15 psi for 60 min efficiently increased the vulnerability of chitin for the myco-chitozymes (Kumar et al., 2020). The activity of chitinase and chitosanase was found elevated up to 320 and 58 Ul^−1^, respectively, while using the pretreated chitin. Furthermore, the synergistic action of both the enzymes (2:1 ratio) on pretreated chitin resulted in a yield of 772 mg L^−1^ of GlcN. Villa-Lerma et al. (2016) demonstrated the pretreatment of chitin using supercritical 1,1,1,2-tetrafluroethane (101°C and 40 bar pressure) followed by rapid depressurization and fibrillation. Following pretreatment, chitin was subjected to enzymatic hydrolysis by β-*N*-acetylhexosaminidase and chitinase derived from *Lecanicillium lecanii*. The result showed the synthesis of highly acetylated COS with F_A_ of 0.45 and DP of 2 to 5. Currently, the utilization of greener chemicals is getting much more attention for pretreatment due to their high digesting capacity and lower environmental toxicity (Mao et al., 2019).

Among the green chemicals, ILs have been extensively studied for the pretreatment of lignocellulosic biomass and their utilization for chitin pretreatment has also been started (Table 4). The dissolution of chitin in a solvent is crucial for the development of value-added biomaterials. However, the high dissolution behavior of chitin in the ILs depended upon the quality of chitin as well as on the dissolution process (Shamshina, 2019). 1-butyl-3-methylimidazolium acetate showed good solvent properties for native chitin (Wu et al., 2008). ILs have also been utilized for the modification of chitin to improve its physicochemical and biological properties. Jaworska et al. (2017) observed that several ILs have an ethyl group as a substituent in the cationic ring that promoted chitin modification. In a study, Berton et al. (2018) suggested that the distribution of product and yield completely relies on the hydrated state and type of substrate chosen for the reaction by conducting a comparative evaluation of enzymatic hydrolysis using chitinase from *Streptomyces griseus*. They used dried shrimp shells (SS), pure commercial grade PG-chitin, chitin hydrogel extracted from SS using IL ([C_2_mim] [OAc] via dissolution and coagulation), and chitin extracted from SS in dried form as substrates. The enzymatic hydrolysis of SS resulted majorly into GlcNAc, whereas (GlcNAc)_2_ has been obtained as a major product in the case of PG-chitin and dried IL-extracted chitin. Contrarily, IL-extracted hydrogel chitin at 25°C yielded (GlcNAc)_2_, which on further increase in temperature resulted in the production of GlcNAc, and finally, at the highest reaction temperature, a minor amount of (GlcNAc)_3_ has been observed as hydrolysis product. Similarly, an acidic IL i.e. 1-(carboxymethyl) pyridinium chloride is synthesized and examined for its unexploited potential to dissolve cellulose, CHS, and chitin (Taheri et al., 2018). The findings of the study suggested that IL treated samples of cellulose and CHS were less crystalline with lower temperatures of degradation. In contrast, chitin, in addition to dissolution, also hydrolyzed to quaternary ammonium CHS.

Moreover, another recent study employed double chitinase, i.e., chip1 and chip2, from *Penibacillus pasadensis* CS0611 on IL pretreated crab shell chitin and resulted in 712.6 mg g^−1^ of COS with DP 2 and 177.1 mg g^−1^ of GlcNAc (Xu et al., 2018). Li et al. (2019) evaluated the effects of IL (1-ethyl-3-methylimidazolium acetate [[C_2_mim][OAc]]) on chitin pretreatment and the yield of subsequent enzymatic hydrolysis utilizing chitinase derived from *Streptomyces albolongus* ATCC27414. The enzymatic hydrolysis of [C_2_mim][OAc] pretreated chitin resulted in the significant production of GlcNAc (175.62 mg g^−1^) and *N, N'-*diacetylchitobiose (341.70 mg g^−1^ chitin) with 61.49% conversion efficiency at 48 h. The authors correlated the apparent performance of enzymes with the evidenced great alleviation in the crystallinity of chitin, high enzyme adsorption ability, and structural changes in porosity and polymer's grain size. Further, they conducted an NMR spectroscopic evaluation of GlcNAc solvation in [C_2_mim][OAc] and confirmed the formation of hydrogen bonding between hydroxyl groups of GlcNAc and cations and anions of IL [C_2_mim][OAc]. Husson et al. (2017) demonstrated the role of two imidazolium-based room temperature ILs (1-ethyl-3-methylimidazolium methylphosphonate, [C_2_mim][MeO(H)PO_2_] and 1-ethyl-3-methylimidazolium acetate, [C_2_mim][OAc]) on the conversion of chitin through sequential and simultaneous strategies. For the enzymatic hydrolysis, commercial chitinases from *Trichoderma viride* or *Streptomyces griseus* have been applied. In the sequential strategy, chitin was pretreated with ILs under mild conditions prior to enzymatic hydrolysis. Compared to [C_2_mim][MeO(H)PO_2_], the IL [C_2_mim][OAc] has resulted in better yield of GlcNAc (185.0 ± 4.0 mg g^−1^ chitin) and (GlcNAc)_2_ (667.60 ± 20.71 mg g^−1^ chitin) following enzymatic hydrolysis by *T. viride* and *S. griseus*, respectively. On the other hand, in the simultaneous strategy, the ILs were used as co-solvents in one plot enzymatic hydrolysis using *S. griseus* yielded 573.72 ± 5.99 mg g^−1^ chitin of (GlcNAc)_2_ with traces of GlcNAc. Furthermore, enzymatic hydrolysis of [C_2_mim][OAc] pretreated chitin by the chitinases concoction from both the organisms resulted in the production of 760.0 ± 0.1 mg g^−1^ chitin. Thus, it can be concluded that the sequential strategy is highly efficient for the conversion of chitin into valuable GlcNAc and (GlcNAc)_2_ (Husson et al., 2017)_._ Qin et al. (2010) performed the dissolution and extraction of crustacean shells to obtain the chitin utilizing ILs ([C_2_mim][OAc]). During dissolution, disruption of hydrogen bonds resulted in the disassembly of the chitin chain, which further gets rearranged into a different arrangement during coagulation and formed amorphous (open hydrated gel type) chitin. The utilization of ILs in the pretreatment of lignocellulosic biomass has been well-explored and showed promising results. However, employment of ILs for chitin pretreatment is relatively less explored. Therefore, given the success of cellulose pretreatment through ILs and some significant studies of the employment of ILs in chitin pretreatment, there is a requirement of further exploration of different ILs for chitin pretreatment to enhance its vulnerability for enzymatic action.

Apart from the pretreatment strategy, the development of synthetic COS can be achieved through the enzymatic glycosylation of a chemically derived COS- oxazoline monomer. The genetically engineered chitinolytic enzymes exhibiting TG activity are used to develop regio- and stereospecific COS. Shoda et al. (2006) synthesized a novel oligosaccharide of N-acetyllactosaminoglycan with DP5 having β-(1 → 4)-β-(1 → 6)-linked repeating units in the main polymeric chain. For this, they used transglycosylating chitinase A1 derived from *Bacillus circulans* WL-12 and an oxazoline derivative of N-acetyllactosamine (LacNAc-oxa) as substrate. Likewise, Yoshida et al. (2012) demonstrated the regio- and stereospecific synthesis of (GlcNAc)7 having β-(1 → 4) linkage by polyaddition of 1,2-oxazoline derivative of (GlcNAc)5 with (GlcNAc)2 through TG activity of chitinase A1 from *B. circulans* WL-12. This chemoenzymatic route for COS production appeared as a promising approach for the commercial-scale production of COS and GlcNAc.

## Purification and Characterization of COS and GlcNAc

The conversion of chitin by the chemical, enzymatic, or synergistic method usually results in a mixture of monomer GlcNAc, oligomers, homologs, and isomers of discrete MW and DP. Thereby, separation and characterization of desired COS and GlcNAc from the reaction mixture is a challenging task. The purification of these products is vital for their characterization to gain an insight about the association between physicochemical properties and biological activities (Liaqat and Eltem, 2018). Moreover, the utilization of COS and GlcNAc in the biomedical and food industries require high purity, quality, and quantification (Xia et al., 2011). Numerous strategies have been reported in the literature for the extraction and purification of COS and GlcNAc, such asgel filtration, ultrafiltration, and nanofiltration (Sørbotten et al., 2005; Lopatin et al., 2009; Dong et al., 2014).

COS can be purified and characterized by employing modern chromatography and spectrometry techniques (Table 3). While purifying derivatized COS, the selection of purification strategy solely relies on the chemical nature of the derivative. Full structural characterization of COS is relatively tedious but pure and small oligomers can be characterized to some extent. Primarily, the analysis of COS is performed through thin layer chromatography (TLC). TLC helps in the separation of a mixture of oligomers based on DP at a relatively low cost, while HP-TLC relies on the DA. However, TLC does not offer an accurate quantitative analysis of the products.

High Performance Liquid chromatography (HPLC) serves as an advancement to TLC as it can smoothly perform the COS analysis based on both DP and DA when coupled with mass spectrometry (Li et al., 2016). GlcNAc can absorb UV light maximum at 204 nm due to the presence of the acetamido group, but sometimes contaminating compounds alter the results by absorbing at a lower wavelength. HPLC can detect only the partial or fully acetylated COS (Seki et al., 2019). Refractive index detectors (RID) are used for COS detection with HPLC. TLC and HPLC were performed to analyze the enzyme-catalyzed hydrolysis products of CHS and N^1^-acetylchitohexaose [(GlcN)_5_-GlcNAc] with a GlcNAc residue at the reducing end (Seki et al., 2019). TLC analysis of CHS illustrated that the enzyme opted as an exo-type pattern of cleavage as (GlcN)_2_ has been obtained as a major product. The observations revealed that (GlcN)_2_ and (GlcN)_3_-GlcNAc have been produced as in substantial quantities and later was further hydrolyzed into GlcN-GlcNAc and (GlcN)_2_. In spite of being widely utilized for COS and GlcNAc characterization, HPLC is linked with several disadvantages viz. low retention time, requirement of a concentrated sample, less suitable for gradient elution, and organic eluent requirement in high amounts (Liaqat and Eltem, 2018). High performance anionic exchange chromatography with pulsed amperometric detection (HPAEC-PAD) has been considered as another promising and efficient tool for the separation of underivatized carbohydrates. HPAEC, by reducing cost and time of the derivatization of COS, offered results with high resolution and sensitivity (Li et al., 2016). Cao et al. (2016) conducted a study on the separation of the underivatized mixture of deacetylated COS (GlcN)_1−6_ using HPAEC-PAD. The researchers used the HPAEC-PAD system comprised of ICS-3000 system, CarboPac-PA100 guard column (4 × 50 mm), and CarboPac-PA100 analytical column (4 × 250 mm). The study reported efficient separation of COS with DP 2 to 6, while GlcN separation was challenging due to the presence of its peak amid (GlcN)_3_ and (GlcN)_4_ peaks.

Capillary electrophoresis (CE) also provided improved purification advancements compared to HPLC. CE offers a high resolution using a small amount of solute and solvent with a comparatively short analysis time (Liaqat and Eltem, 2018). The separation of COS proceeds efficiently in aqueous acidic solution as they can adsorb on negatively charged surfaces, such asfused silica capillaries in acidic solution (Sarbu and Zamfir, 2018). Hattori et al. (2012) demonstrated the separation of COS through simple CE by using *N*-trimethoxypropyl- *N, N*, -Ntrimethylammonium chloride coated positively charged capillary. However, the process of derivatization and utilization of highly expensive materials make the CE economically less efficient. Recently, nuclear magnetic resonance (NMR) spectroscopy is considered to be one of the best techniques for the structural analysis of COS and to determine their degree of deacetylation (DD). NMR provides the complete spectra of reducing sugars, non-reducing sugars, and disparities in its nearest neighbors. Jiang et al. (2017) reported two methods for the analysis of DD in COS: the first method involved acid-base titration with bromocresol green indicator, while the second included first-order derivative UV spectrophotometric method. The accuracy in the results of both the techniques was verified by comparing the results obtained with ^1^H NMR spectroscopy. Another study prepared COS with two different processes: the first was completed solely through enzymatic hydrolysis employing chitosanase, while the second involved chemical hydrolysis followed by an enzymatic one (Sánchez et al., 2017). The study analyzed COS produced through ^1^H NMR spectroscopy and MALDI-TOF-MS and observed that COS with 63% fully deacetylated sequences were generated in the second process. Cao et al. (2019) confirmed the synthesis of Naringin and Chitooligosaccharide (Nari-COS) complex by using scanning electron microscopy (SEM) and ^1^H NMR. However, COS analysis through NMR spectroscopy requires more development as it is limited to only low DP COS (< (GlcNAc)_5_) along with the requirement of the concentrated sample.

Currently, MALDI-TOF-MS emerged as the most appropriate technique to analyze biomolecules i.e., lipids, saccharides, peptides, and other organic macromolecules through the ionic mass and charge. MALDI-TOF-MS involves the soft-ionization process, which leads to lesser or no fragmentation of analytes that offers the identification of molecular ions of analytes even within complex mixtures such as COS (Liaqat and Eltem, 2018). Santos-Moriano et al. (2019) performed controlled enzymatic hydrolysis of CHS or chitin and obtained three types of COS viz. fully acetylated (*fa*COS), fully deacetylated (*fd*COS), and partially acetylated (*pa*COS). The study employed HPAEC-PAD and MALDI-TOF-MS to determine the chemical composition of obtained COS (Santos-Moriano et al., 2019). Despite having several advantages over other COS characterization techniques, MALDI-TOF-MS also holds a few drawbacks, such as havinga lower mass limit (not <500 Da) of the sample to be analyzed (Li et al., 2016). However, the recent development in the purification and characterization of COS and GlcNAc have increased the medicinal value of these bioactive molecules. This has resulted in the further improvement of the biological activities of COS and GlcNAc through engineering/derivatization.

## COS and GlcNAc Derivatization/Engineering for Improving Biological Activities

**Figure 5:**
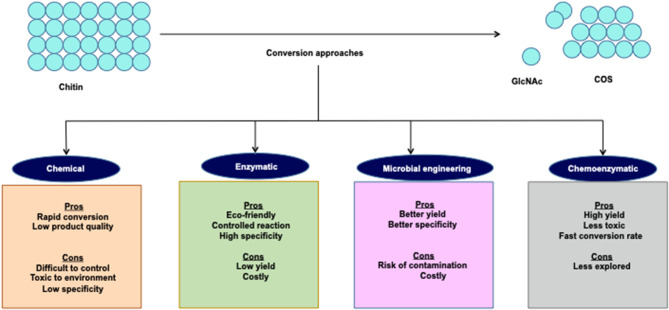
Synthesis of COS derivative.

COS and GlcNAc embrace several biological activities and have diverse applications in several fields viz. food, agriculture, pharmaceuticals, biomedical, and cosmetics, owing to their biocompatibility, biodegradability, and renewable production. Currently, engineering of the functionalized COS and GlcNAc derivatives with enhanced biological activities have gained worldwide attention due to remarkable applications in medicine (Figure 5). For example, phenolic compounds conjugated COS have significant medical applications (Liaqat and Eltem, 2018). Numerous recent studies reported the improvement of COS's biological activities through derivatization (Table 5). Several derivatives of COS and GlcNAc conjugated with aminoethyl, phenolic compounds, gallic acid, carboxyl group, and sulfate have been reported so far. Ngo et al. (2019) formed aminoethyl (AE) conjugated derivative of COS by replacing the hydroxyl group present at C-6 position with AE. The aminoethylation reactivity was found to be highest at the C-6 position. The aminoethyl COS (AE-COS) was readily soluble in water and further examined for its potential to inhibit angiotensin I converting enzyme (ACE). The findings revealed that at 2.5 mg mL^−1^ it displayed 89.3% ACE inhibition through AE-COS (2.5 mg mL^−1^) with IC_50_ value of 0.8017 mg mL^−1^. AE-COS was investigated to assess the antiproliferative effect on cell invasion of human fibrosarcoma cells (Hong et al., 2016). Firstly, the impact of AE-COS on cell viability was observed, followed by the utilization of gelatin zymography and western blot to analyze the inhibitory effects of AE-COS on the activity and expression levels of matrix metalloproteinases (MMP) related to the invasion of cancer cells, i.e., MMP-2 and MMP-9. AE-COS downregulated the expression of MMP-9 at a concentration >20 g ml^−1^, whereas an expression of p50 was reduced when applied at <20 g ml^−1^, concentration (Hong et al., 2016). Similarly, the antiproliferative activity of AE-COS on AGS human gastric adenocarcinoma cells was also evaluated. Three amino derivatized COS, i.e., aminoethyl-chitooligosaccharide (AE-COS), dimethyl aminoethyl-chitooligosaccharide (DMAE-COS), and diethyl aminoethyl-chitooligosaccharide (DEAE-COS) were prepared and confirmed through IR spectra. The amino derivatized COS showed antiproliferative potential on AGS human gastric adenocarcinoma cells and AE-COS and DEAE-COS exhibited higher apoptotic activity than DMAE-COS (Karagozlu et al., 2010). AE-COS has been observed to decrease the cell viability of human lung A549 cancer cells to 32 ± 1.3% at a concentration of 500 μg mL^−1^ (detection: MTT assay) (Ngo et al., 2019). AE-COS (500 μg mL^−1^) has appreciably blocked the expression of cyclooxygenase-2 (COX-2) and Bcl-2 while upregulating the expression of apoptotic proteins caspase-3 and 9 in A549 cancer cells. AE-COS is considered as a potent candidate to be utilized as a chemotherapeutic agent in a dose-dependent manner for cancer treatment.

**Table 5:** Biological activities of some COS derivatives.

**COS derivative**	**Biological activity**	**Activity against**	**References**
4-hydroxybenzyl COS	Antioxidant	Chang liver cells	Trinh et al., 2014
Gallic acid-COS	Anti-proliferative	Human gastric cancer cells	Ryu et al., 2017
Geraniol-COS	Antibacterial	*Staphlococcus aureus* *Esherichia coli*	Yue et al., 2017
Gallic acid-COS	Antioxidant Antibacterial	*Propionibacterium acnes*	Park et al., 2018
Carboxylated-COS	Anti-melanogenic COS	Melanin Pigmentation	Zhen et al., 2019
Aminourea-COS	Antifungal	*Fusarium solani Verticillium albo-atrum* *Phytophthora capsici*	Zhu et al., 2019

The carboxylated COS (C-COS) can be synthesized by incorporating the carboxyl group to the amino position at the C-2 of pyranose unit. Rajapakse et al. (2006) synthesized three different C-COS-(1/-2/-3) using succinic anhydride and evaluated their inhibitory potential on MMP-9 expression on human fibrosarcoma cell line (HT1080). The C-COS were distinct to each other based on the increase in the mole ratio of COS/succinic anhydride. The MTT assay of all three C-COS revealed that they do not exhibit cytotoxicity even at a concentration of 500 μg mL^−1^. Further, the researchers confirmed the dose-dependent inhibition (100 μg mL^−1^ of C-COS-1/2/3 led to 50% inhibition) of MMP-9 mediated gelatinolytic activities in HT1080 cells through zymography. The increment in the substitution degree of C-COS was directly proportional to the MMP-9 inhibition. Furthermore, C-COS-3 blocked the MMP-9 expression at 100 μg mL^−1^ significantly by blocking the transcription of activator protein −1 that further inhibited the invasiveness of HT1080 cells. However, C-COS did not show any considerable effect on the inhibition of another transcription factor of MMP-9 expression i.e., NF-κB. Rajapakse et al. (2007) also examined the anti-oxidative potential of C-COS in human (HL60) and mouse macrophages (RAW264.7 cells). DPPP oxide fluorescence assays and thiobarbituric acid reactive substance (TBARS) assays were performed using both C-COS and COS for the inhibition of cell membrane lipid peroxidation. The results showed that at 1,000 μg mL^−1^ both C-COS and COS inhibited DPPP oxide fluorescence intensity by 63 and 50%, respectively. In contrast, TBARS has been reduced to 75% using C-COS (1,000 μg mL^−1^) with no significant inhibitory effects of COS. In HL60 cells, C-COS has been observed to reduce myeloperoxidase activity by 43% at 1,000 μg mL^−1^. Furthermore, C-COS was also found to have the ability to inhibit the formation of reactive oxygen species (ROS), such as superoxide radicals H_2_O_2_ and HOCl, more than the COS. This was confirmed by Direct radical scavenging studies carried out with the DCFH-DA fluorescence probe.

3, 4, 5-trihydroxybenzoic acid, commonly known as Gallic acid, is a well-known secondary polyphenolic metabolite that exhibited antioxidant, antimicrobial, anti-inflammatory, anticarcinogenic, antiangiogenic, and antimutagenic activities (Choubey et al., 2015). Gallic acid conjugated COS were synthesized by the covalent linking of gallic acid to COS via carbodiimide with a subsequent assessment of the enhanced cellular antioxidant activity. The TBARS and DPPP assays were performed to check the lipid peroxidase inhibitory potential of G-COS and a reduction was observed in TBARS by 80% and in lipid peroxide level by 62% at 100 μg mL^−1^ in a dose-dependent manner. COS alleviated 50% of DPPP oxide fluorescence intensity when applied at a concentration of 100 μg mL^−1^. They demonstrated the intracellular radical scavenging potential of G-COS through the DCFH-DA method. Further, G-COS (100 μg mL^−1^) inhibited membrane protein oxidation and radical-mediated DNA damage in a dose-dependent manner in RAW264.7 cells by 83 and 90%, respectively. Contrarily, at similar concentrations, COS (100 μg mL^−1^) has been observed to prevent only 20% of the radical-mediated DNA damage. Moreover, G-COS elevated the level of intracellular antioxidant enzymes [superoxide dismutase (SOD) and glutathione (GSH)] while blocking the activation and expression of NF-κB in H_2_O_2_ induced RAW264.7 cells (Ngo et al., 2011b). Similar results were obtained with SW1353 cells in a study conducted by Ngo et al. (2011a). The study illustrated G-COS as an efficient free radicals scavenger and showed that it also prevents oxidative damage in DNA, proteins, and lipids of SW1353 cells with increasing levels of antioxidant enzymes. The findings indicated that G-COS can be utilized as leading antioxidants in the food and pharmaceutical industries. G-COS was also found to exhibit anti-inflammatory activities in human lung epithelial A549 cells. In this study, G-COS (200 μg mL^−1^) prevented the H_2_O_2_-induced DNA damage and ROS production in A549 cells. G-COS displayed 70% DPPH radical scavenging activity at a concentration of 200 μg mL^−1^, whereas COS showed only 20%. On the contrary, downregulation of cyclooxygenase-2 (COX-2) expression was evidenced along with the reduction in production of PGE2 from LPS-stimulated A549 cells from 64 ± 4 −30 ± 4 pg ml^−1^ at 200 μg mL^−1^ of G -COS. The production of cytokines (IL-8 and TNF-α) was also inhibited in a dose-dependent manner by G-COS. The control or LPS treated cells had 3,124 ± 25 pg ml^−1^ and 45± 1 pg ml^−1^ of IL-8 and TNF-α, but G-COS curtailed it to 1,246 ± 15 pg ml^−1^ and 11 ± 3 pg ml^−1^, respectively (Vo et al., 2017). The results highly enlighten the anti-inflammatory and anti-oxidative potential of G-COS.

Sulfated COS (S-COS) was synthesized with different degrees of substitution (DS) for the enhancement of its water-solubility as well as biological activities. S-COS holds the antioxidant activity as it protected the MIN6 cells from H_2_O_2_ induced dysfunction. S-COS-I (DS-0.8) and S-COS-II (DS-1.9) enhanced cell viability in a dose-dependent manner and at the highest concentration (500 μg mL^−1^), it reached up to 91.7 ± 7% and 95.6 ± 5.3%, respectively. Observations suggested that S-COS-I and S-COS-II were successful in suppressing the expression of H_2_O_2_ induced Bax mRNA and Caspase-3 mRNA in H_2_O_2_ induced MIN6 cells to 1.04 ± 0.08 and 0.79 ± 0.02, respectively. NF-κB/p65 activation and upregulation of Bcl-2 mRNA expression has also been reported (Lu et al., 2013). Acidic fibroblast growth factor (aFGF) plays a vital role in the growth and survival of neurons and is a possible treatment for peripheral nerve injury. Heparin-like properties of S-COS enabled them to improve the biological activities of aFGF. Na_2_S_2_O_4_ hypoxia/reoxygenation injury was induced in RSC96 cells against which the protective effects of S-COS with or without aFGF were investigated. Cell viability and cytotoxicity was assessed by MTT assay and lactate dehydrogenase (LDH) release into the culture medium, respectively. The results showed that COS-S improved the protective effects of aFGF on nerve repair and restoration of function in rats with sciatic nerve injury (Liu et al., 2019).

Phenolic acids belong to the broadly distributed plant non-flavonoid phenolic compounds and are anti-oxidative. COS conjugated with eight different types of phenolic acids (namely, caffeic acid, hydroxybenzoic acid, p-coumaric acid, vanillic acid, ferulic acid, syringic acid, sinapinic acid, and protocatechuic acid) were synthesized by Eom et al. (2012) and the conjugates were formed via amide coupling reaction. The ability of phenolic acids to donate H-atom has enhanced the antioxidative nature of conjugated COS. The characterization of synthesized conjugates was performed by UV, FTIR, and ^1^H NMR data. Caffeic acid conjugated COS and protocatechuic acid conjugated COS have shown a comparatively higher reducing power and radical scavenging (NO and DPPH) activity as compared to COS and other derivatives (Eom et al., 2013). Therefore, caffeic acid conjugated COS can be utilized as an antioxidant compound, that was synthesized by using hydroxyl cinnamic acid and hydroxyl benzoic acid for conjugation with COS. These conjugates were evaluated for their inhibitory activities against the β-site amyloid precursor protein (APP)-cleaving enzyme (BACE). BACE plays a critical role in reducing the levels of Aβ amyloid peptide in Alzheimer's disease (AD). The results showed that caffeic acid conjugated COS derivative has significantly inhibited the BACE and reduced the risk of AD (Eom et al., 2013). Therefore, engineering of COS and GlcNAc derivatives can significantly improve their biological activities. Hence, further research and development in the derivatization can result in the augmentation of biomedicinal applications of COS and GlcNAc.

## Conclusions

COS and GlcNAc possess remarkable biological activities and are in tremendous need for advancements for their use in biomedicine. However, despite being thoroughly studied, the production of COS and GlcNAc through chemical and enzymatic approaches has not attained the desired level for large-scale production of well-defined COS in terms of DP, MW, PD, and PA. Chemical methods led to the generation of toxic waste along with modified products, while the enzymatic methods produce well-defined COS but in a very low yield. Synergy of the chemical and enzymatic process seems to be effective in improving COS and GlcNAc production. Moreover, the utilization of ILs for substrate pretreatment, followed by enzymatic hydrolysis, showed high potentiality for development of an efficient and environmentally-friendly method. Thus, investigation is needed to develop controlled chemoenzymatic processes for the generation of high COS and GlcNAc titres. Additionally, the engineering of COS and GlcNAc and derivatization by employing strategies, i.e., TG and genetic recombination approaches, can further enhance their biological activities. In the light of research done so far, it can be concluded that despite numerous studies for the development of efficient COS and GlcNAc production processes, there is still a need to advance chemoenzymatic approaches to augment the COS and GlcNAc production levels. Moreover, the engineering and derivatization of COS can further improve their biological functions and so also need detailed molecular investigation.

## Author Contributions

MK conceived and wrote the review article. MR performed a literature survey and helped in preparing the manuscript. TS performed sketch work. VV proofread and give valuable advice on the content of the review. NP supervised, conceptualized, wrote, and edited the review article. All authors contributed to the article and approved the submitted version.

## Conflict of Interest

The authors declare that the research was conducted in the absence of any commercial or financial relationships that could be construed as a potential conflict of interest.
